# Efficacy and safety of molnupiravir in patients with Omicron variant vaccine breakthrough COVID-19 infection: a randomized, controlled trial

**DOI:** 10.3389/fphar.2023.1197671

**Published:** 2023-11-16

**Authors:** Yayun Liu, Shiyong Fan, Aijing Xu, Lingling Ge, Xinyu Wang, Xu Dong, Mingxiao Xu, Wenhan Fan, Wu Zhong, Xuesong Liang

**Affiliations:** ^1^ Department of Infectious Diseases, First Hospital of Navy Military Medical University, Shanghai, China; ^2^ National Engineering Research Center for the Emergency Drug, Beijing Institute of Pharmacology and Toxicology, Beijing, China

**Keywords:** antiviral drugs, molnupiravir, severe acute respiratory syndrome coronavirus 2, Omicron variant, vaccine breakthrough infection

## Abstract

**Introduction:** Randomized, controlled trials of molnupiravir in real-world use during the Omicron wave are scarce. The frequency of hospitalization and death is low, so further research is needed to confirm the virological efficacy of molnupiravir.

**Methods:** A single-center, randomized, controlled clinical trial was conducted, and 111 hospitalized coronavirus disease 2019 (COVID-19) patients were randomly assigned at a ratio of 1:1. Fifty-three patients in the molnupiravir group were administered 800 mg of molnupiravir twice daily for 5 days in addition to the standard therapy, and 58 patients in the control group only received the standard therapy in accordance with local guidelines. The antiviral effect and adverse events were evaluated during the follow-up.

**Results:** The median viral clearance time in the molnupiravir group was significantly shorter than that in the control group (*p* = 0.003). Furthermore, patients who started molnupiravir therapy within 3 days had significantly shorter viral clearance time than the controls (*p* = 0.003). In the vaccinated subgroup, molnupiravir therapy was also associated with a shorter viral clearance time (*p* = 0.003). A total of three adverse events, which were minor, were reported in the molnupiravir group. One of the patients had mild liver function abnormalities, and all of them were resolved without intervention. However, the remission time was similar between the two tested groups.

**Conclusion:** Molnupiravir exhibited good viral replication inhibitor efficacy in patients with Omicron variant vaccine breakthrough COVID-19 infection.

**Clinical Trial Registration:** [https://www.chictr.org.cn/], identifier [ChiCTR2200059796].

## Introduction

The coronavirus disease 2019 (COVID-19) pandemic, which was caused by severe acute respiratory syndrome coronavirus 2 (SARS-CoV-2), has been a major challenge for the medical community. SARS-CoV-2 has evolved continuously since its emergence. Currently, the Omicron variant is the dominant lineage worldwide. Compared with other variants, Omicron has a greater ability to evade antibody neutralization developed by infections with previous variants or by vaccines owing to high mutations in the spike protein, which is the target of most COVID-19 vaccines and therapeutic antibodies (Grein et al., 2020; Dejnirattisai et al., 2022; Guo et al., 2022; Islam et al., 2022; Mohsin and Mahmud, 2022; Qin et al., 2022). Fortunately, oral antiviral drugs are unlikely to lose activity against the variants because the targets (RNA-dependent RNA polymerase, RdRp; main protease) are more conserved than the spike protein (Udwadia et al., 2021; Cao et al., 2022; Shen et al., 2022; Vangeel et al., 2022). Vangeel et al. (2022) and Takashita et al. (2022) separately confirmed that molnupiravir retains activity against Omicron *in vitro*. However, clinical research on the efficacy of molnupiravir in the Omicron variant, especially in patients with vaccine breakthrough COVID-19 infection, is scarce.

Molnupiravir, a novel oral antiviral drug, has been approved for use in the treatment of COVID-19 in multiple countries for nonhospitalized adult patients with mild-to-moderate COVID-19 (Fischer et al., 2022; Jayk Bernal et al., 2022). The approval of molnupiravir was mainly based on the result of a phase 3, double-blind, randomized, placebo-controlled trial (MOVe-OUT, NCT04575597, MK-4482-002). In the aforementioned trial, the three most common SARS-CoV-2 variants observed in the participants were B.1.617.2 (Delta; 58.1%), B.1.621 (Mu; 20.5%), and P.1 (Gamma; 10.7%) (Jayk Bernal et al., 2022).

Unlike the findings from the MOVe-OUT clinical trial, patients’ vaccination status and the circulating variant of concern have changed because most of the patients have been vaccinated against COVID-19, and the Omicron variant has been the dominant lineage worldwide since 2022. Evaluating the efficacy of molnupiravir during the Omicron wave in real-world settings, where most people are vaccinated, is important.

We conducted a search in PubMed for studies published before 1 March 2023 using the terms “SARS-CoV-2 OR COVID-19” AND “molnupiravir OR EIDD-2801” AND “Real-world.” To the best of our knowledge, several real-word clinical studies had been conducted to evaluate the efficacy of molnupiravir on different SARS-CoV-2 lineages, and the characteristics of these studies are shown in Supplementary Table S1 (Atluri et al., 2022; Flisiak et al., 2022; Liu et al., 2022; Lupia et al., 2022; Pontolillo et al., 2022; Streinu-Cercel et al., 2022; Suzuki et al., 2022; Wong et al., 2022; Butler et al., 2023). Most of the studies were retrospective cohort study (Atluri et al., 2022; Flisiak et al., 2022; Liu et al., 2022; Lupia et al., 2022; Pontolillo et al., 2022; Streinu-Cercel et al., 2022; Suzuki et al., 2022; Wong et al., 2022), and the main virus lineage was the non-Omicron lineage. For instance, in AGILE CST-2, a randomized, placebo-controlled, double-blind, phase 2 trial conducted in UK, the participants were infected by Delta (B.1.617.2; 72 [40%] of 180), Alpha (B.1.1.7; 37 [21%]), Omicron (B.1.1.529; 38 [21%]), and EU1 (B.1.177; 28 [16%]) (Khoo et al., 2023).

Randomized, controlled trials of molnupiravir in real-world settings during the Omicron wave are scarce. Here, we report a single-center, randomized, controlled trial of molnupiravir in a population infected with the SARS-CoV-2 Omicron variant during the COVID-19 pandemic wave in Shanghai, China, from 26 March 2022 to 31 May 2022.

## Methods

### Trial design and participants

A single-center, randomized, controlled trial evaluating the safety and efficacy of molnupiravir in a population infected with the SARS-CoV-2 Omicron variant was conducted in Changhai Hospital (Shanghai, China) between 26 March 2022 and 31 May 2022. The study was reviewed and approved by the Local Ethical Committee (CHEC 2022-055), registered at chictr.org.cn (ChiCTR2200059796), and performed in accordance with the Helsinki Declaration of 1964 and its later amendments. Informed consent was obtained from all participants before enrollment and drug administration.

The trial included eligible participants who were between 18 and 70 years of age and diagnosed with SARS-CoV-2 infection confirmed via real-time RT-PCR. The exclusion criteria were as follows: 1) diagnosed with recurring COVID-19 via PCR; 2) received convalescent plasma for COVID-19, monoclonal antibody to SARS-CoV-2, or other antiviral treatment before admission; 3) symptom onset or first positive test prior to admission longer than 5 days; 4) had a history of severe allergic reactions, including generalized urticaria, angioedema, and hypersensitivity reactions; 5) enrolled in other clinical trials; 6) refused to participate in the trial; and 7) lactating, pregnant, or refused contraception during treatment.

### Procedures

The participants were randomly assigned to either the molnupiravir group or the control group at a ratio of 1:1 by using a random number table. Patients in the molnupiravir group were administered 800 mg of molnupiravir (Lot #0000033094, provided by National Engineering Research Center for the Emergency Drug, Beijing, China) twice daily for 5 days in addition to standard remission treatment, whereas patients in the control group only received the standard remission treatment. The standard remission treatment included nonsteroidal, anti-inflammatory drug, paracetamol (if the body temperature ≥38 °C), and a cough mixture, licorice mixture, a traditional Chinese medicine.

Participant details about demographic characteristics, medical history, and vaccination status against COVID-19 were collected at admission. Vital signs and disease conditions were monitored by clinicians every day. Inflammation index, white blood cell counts, lymphocyte count, c-reactive protein (CRP), interleukin (IL)-6, D-dimer, and other biochemical indices, such as liver and kidney function indicators, were monitored every 3 days. All patients underwent a chest CT scan at admission.

Adverse events were assessed during the treatment period and for 28 days since the enrollment of the trial.

### Virus detection and isolation

The SARS-CoV-2 viral load (VL) was detected and quantified by real-time RT-PCR (Novel Coronavirus (2019-nCoV) Real Time RT-PCR Kit, Cat.#:RR-0479-02, Liferiver Bio-Tech (China) Corp., Shanghai) on alternate days since hospital admission. The PCR-positive throat swab sample from the patients was used to isolate SARS-CoV-2 via Vero-E6 cells. In brief, the PCR-positive throat swab sample from the patient was diluted at 1:1 with 2X double-antibody DMEM before it was added to the Vero-E6 cells. The supernatant was discarded after incubation at 37°C for 2 h. After washing with pre-cooled PBS three times, fresh DMEM was added. The cells were incubated at 37°C and observed daily for cytopathogenic effects. The supernatant was collected for sequencing after the cytopathogenic effects were observed. The virus isolation was sequenced and submitted to GenBank.

A cycle threshold (ct) value > 35 for both ORF1ab and N genes was considered to indicate negativity. Two consecutive negative tests at least 24 h apart were defined as nucleic acid test negative conversion or undetectable VL.

### Endpoints

#### Efficacy endpoint

The primary efficacy endpoint was defined as the time to viral clearance. Undetectable VL time or viral clearance time was defined as the first positive nucleic acid test time to the first negative test time (in two consecutive tests).

The secondary efficacy endpoint was the proportion of patients who tested negative in the nucleic acid test or had undetectable VL on days 5, 7, 11, and 13 and VL change during treatment duration.

#### Safety endpoint

The primary safety endpoint included adverse events of grade 3 or higher after treatment or events leading to early discontinuation of treatment.

The secondary safety endpoint included all adverse events during and post-drug administration.

#### Statistical analysis

In anticipation of up to 10% missing data, 121 participants (approximately 61 each for the molnupiravir group and the control group) provided 80% power to detect a risk difference (molnupiravir−control) of −15% in the primary endpoint using a type I error rate (*α*) of 0.025. Continuous variables were expressed as median (range) or mean (standard deviation) and compared with the nonparametric test results. Categorical variables were expressed as numbers (%) and compared using the χ^2^ test or Fisher’s exact tests. The time of viral RNA clearance was summarized using the Kaplan–Meier methodology, and the log-rank test was used for comparison of the efficacy between treatments. The percentage of patients who were negative for viral RNA was summarized and compared between groups through Fisher’s exact test. A *p*-value <0.05 was considered statistically significant.

## Results

### Participants’ demographic and clinical characteristics

**FIGURE 1:**
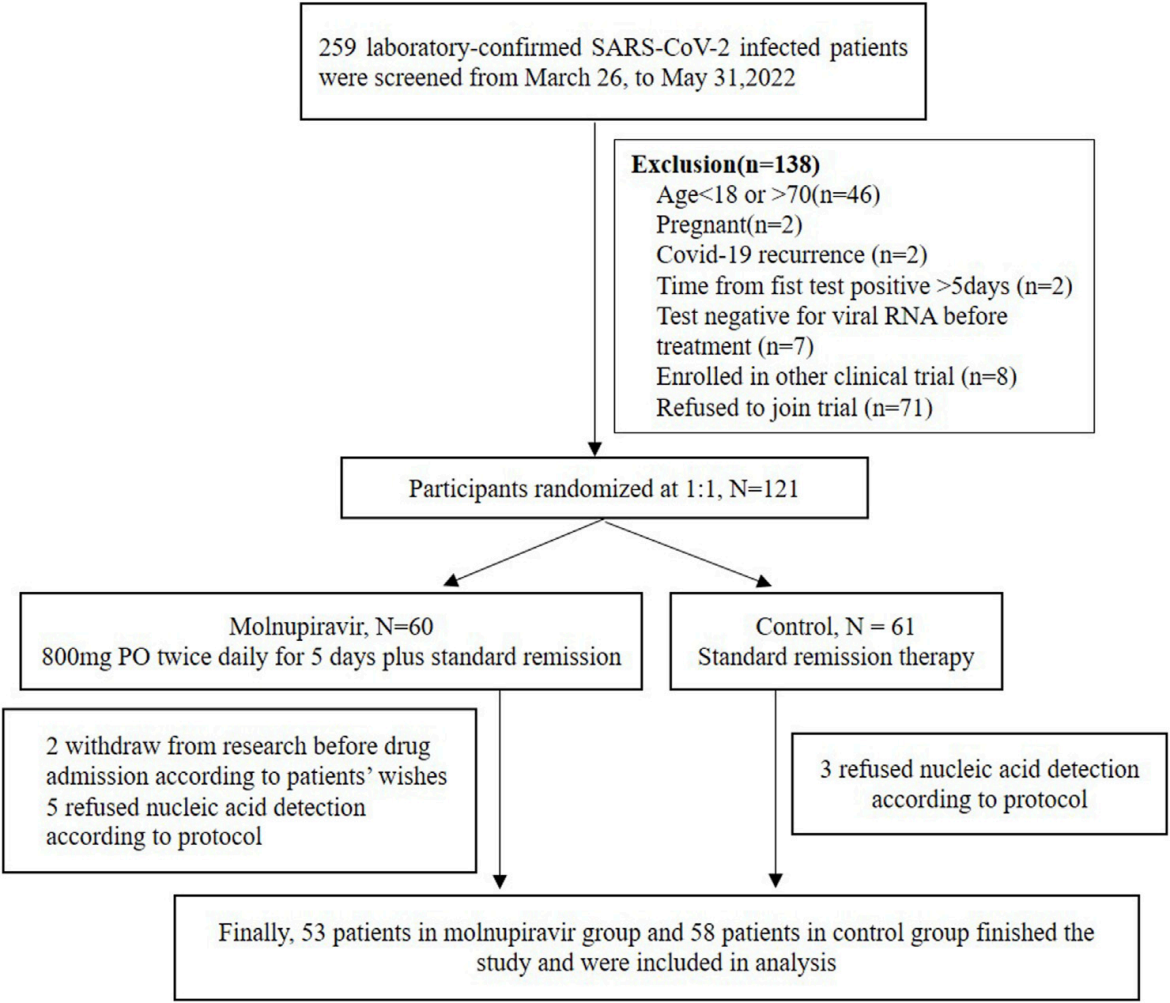
CONSORT diagram for this trial.

A total of 259 hospitalized laboratory-confirmed SARS-CoV-2-infected patients were screened. Among them, 121 patients were randomized: 60 were randomized to the molnupiravir group and 61 to the control group. Ten of these participants refused to undergo the viral nucleic acid test according to research protocol or asked to withdraw from the trial. Finally, 53 patients in the molnupiravir group and 58 patients in the control group completed the entire therapy and follow-up and were included in efficacy and safety analyses (Figure 1). All of these patients completed at least 3-time point VL test and 1-time point virus isolation and genome sequencing. We finally isolated 11 strains of the virus, and all of them belonged to the Omicron variant. The sequence was submitted to PubMed GenBank (ON965380; ON965371; ON965362; ON965361).

Baseline characteristics were generally comparable between the two groups; however, the molnupiravir group had a higher initial SARS-CoV-2 RNA level for the nucleocapsid protein and a lower proportion of patients whose therapy time from the first positive test was less than 3 days (Table 1).

**TABLE 1:** Baseline characteristics.

Characteristic	Molnupiravir (n = 53)	Control (n = 58)	*p*-value
Age, mean ± SD, years	39.1 ± 1.88	35.1 ± 1.72	0.119
Male, n (%)	22 (41.5)	27 (50.9)	0.436
BMI, mean ± SD, (kg/m^2^)	22.4 ± 0.41	22.9 ± 0.35	0.436
Ever medical history, n (%)			
Hypertension	6 (11.3)	3 (5.2)	0.402
Diabetes	2 (3.8)	0	0.428
Others	5 (9.6)	2 (3.4)	0.351
Vaccination status against COVID-19, n (%)			
Unvaccinated	6 (11.3)	3 (5.2)	0.465
Base vaccinated	9 (17.0)	12 (20.7)	
Booster vaccinated	38 (71.7)	43 (74.1)	
Clinical type, n(%)			
Asymptomatic	7 (13.2)	13 (22.4)	0.207
Mild/moderate	46 (86.8)	45 (77.6)	
Symptom onset, n (%)			
Fever	24 (45.3)	24 (41.4)	0.678
Cough	20 (37.7)	22 (37.9)	0.983
Pharyngeal symptoms	23 (43.4)	22 (37.9)	0.558
Others	18 (34.0)	12 (20.7)	0.174
>1 symptoms	27 (50.9)	26 (44.8)	0.519
Initial SARS-CoV-2 RNA level (log_2_ ct value)			
ORF1ab, mean ± SD	4.34 ± 0.04	4.38 ± 0.04	0.531
N, mean ± SD	4.12 ± 0.05	4.27 ± 0.04	0.036
Administration time since the first positive test			
<3 days, n (%)	32 (60.4)	47 (81.0)	0.029
≥3 days, n (%)	21 (39.6)	11 (19.0)	
Median (range), days	2 (0–7)	2 (1–8)	0.858
Mean ± SD, days	2.28 ± 0.195	2.26 ± 0.138	0.918

SD, standard deviation; viral RNA test cycle threshold (ct) value was expressed as log_2_ (ct value); continuous variables were compared using a non-parametric test, and categorical variables were compared using the χ^2^ test or Fisher’s exact tests.

^a^ Fully vaccinated patients were defined as those with at least two doses of Comirnaty or three doses of CoronaVac.

SD, standard deviation; BMI, body mass index; COVID-19, Coronavirus disease 2019; SARS-CoV-2, severe acute respiratory syndrome coronavirus 2.

Of these participants, 91.89% received base vaccination or booster against COVID-19 (Comirnaty or CoronaVac), and the vaccinated rate of the molnupiravir and control groups was 88.7% and 94.8%, respectively (Table 1). The vaccination scheme was homologous vaccine reinforcement. The last dose date to the confirmed SARS-CoV-2 infection of patients who received the booster was shorter than 6 months, but that of patients who only received base vaccination was longer than 6 months. The vaccination status between the two groups was similar (Table 1).

The most common onset symptoms were fever, cough, and pharyngeal discomfort; nearly half of these participants experienced more than one symptom. There was no difference in the clinical manifestation and diagnosis between the two groups (Table 1).

### Clinical outcomes

Overall, all participants experienced a mild clinical process. All laboratory test results of these patients, including inflammatory indicators, such as CRP, IL-6, and lymphocyte count, returned to the normal range at discharge, regardless of whether they received molnupiravir or not. There was no significant difference in the median remission time of clinical symptoms between the two groups (1.5 days vs 3.0 days, *p* = 0.851).

### Antiviral efficacy

The primary endpoint of this study was the viral clearance time in nasopharyngeal swabs via the real-time RT-PCR test. Overall, compared with the patients in the control group, those in the molnupiravir group experienced faster viral clearance. The median time till viral clearance of SARS-CoV-2 in the molnupiravir group was significantly shorter than that in the control group (Table 2).

**TABLE 2:** Antiviral efficacy of molnupiravir.

Variable	Therapy strategy categories		
	Molnupiravir (n = 53)	Control (n = 58)	P* value
Median viral shedding time (days), median (IQR)	10 (6–11.0)	10.5 (8.0–14.0)	0.003
Mean viral shedding time (days), mean ± SD	9.26 ± 3.89	11.84 ± 4.83	0.002
D5 undetectable VL, n (%)	10 (18.9)	0 0)	0.002
D7 undetectable VL, n (%)	21 (39.6)	9 (15.5)	0.008
D10 undetectable VL, n (%)	38 (71.7)	29 (50.0)	0.032
D14 undetectable VL, n (%)	48 (90.6)	45 (77.6)	0.064

VL, viral load; IQR, interquartile range; SD, standard deviation; D5, day 5; D7, day 7; D10, day 10; D14, day 14; the *p*-value refers to the comparison between the molnupiravir and control groups. *p* < 0.05 means significant difference.

**FIGURE 2:**
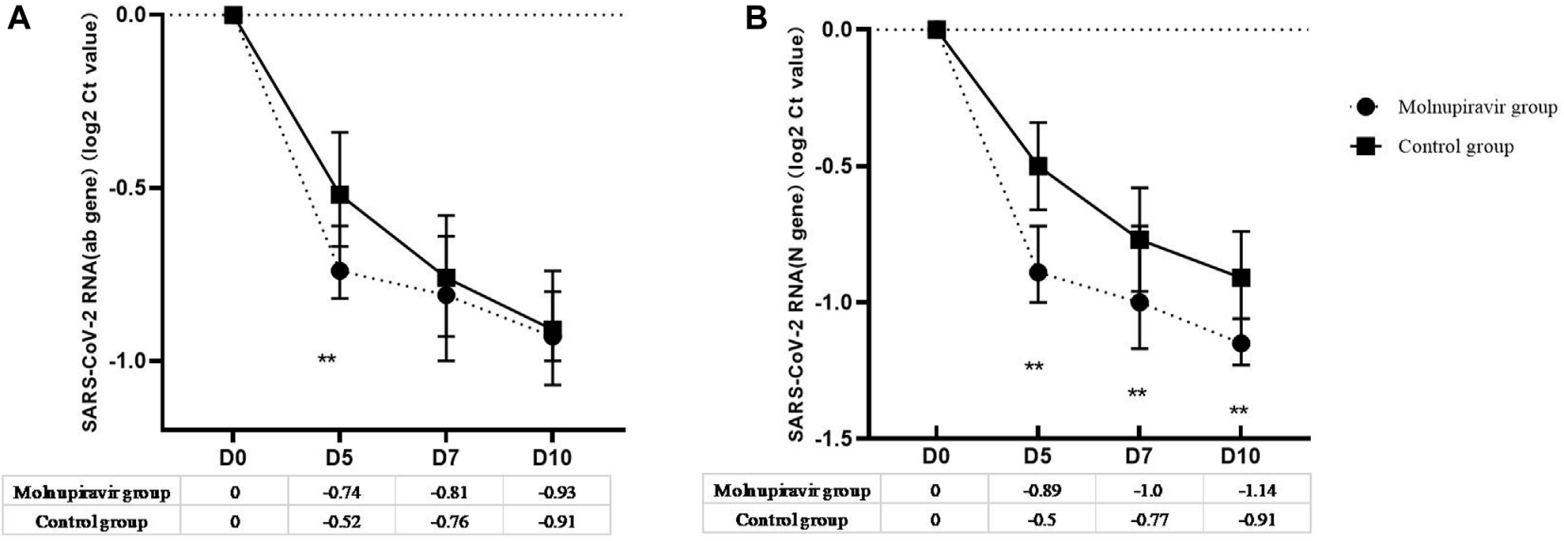
Viral load change from baseline after treatment. **(A)** ORF1ab gene; **(B)** N gene. ** means *p*-value < 0.05.

The secondary efficacy endpoint was the VL change and proportion of patients who tested negative in the nucleic acid test or had undetectable VL on days 5, 7, 10, and 14 (Figure 2; Table 2). On day 5 of treatment, patients in the molnupiravir group achieved a sharp VL decrease for ORF1ab (0.74 log_2_ ct value) and N (0.89 log_2_ ct value) genes, and 18.9% patients achieved viral clearance, both of which were significantly higher than those in the control group (*p* = 0.002). On days 7 and 10 of treatment, the undetectable VL rate of the molnupiravir group increased to 39.6% and 71.7%, respectively, which were significantly higher than those of the control group (15.5% vs 50.0%; *p* = 0.008, 0.032; Figure 2; Table 2).

**FIGURE 3:**
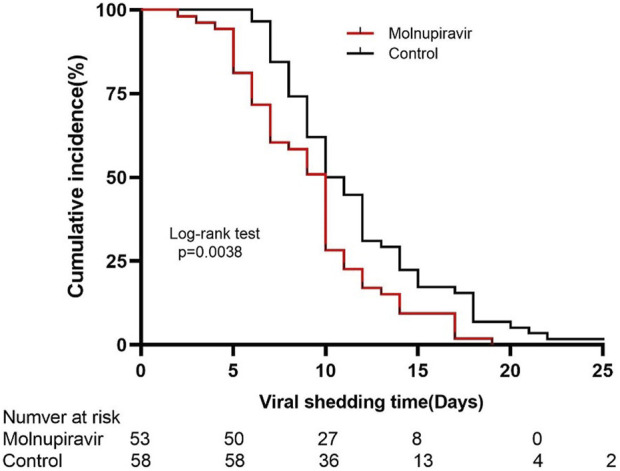
Kaplan–Meier estimate of the time from the first positive testing to the first day of nucleic acid ct value ≥ 35 for both ORF1ab and N genes.

The Kaplan–Meier survival curve was used to analyze the cumulative incidence of the negative nucleic acid test of the two groups. The cumulative nucleic acid negative conversion rate of the molnupiravir group was significantly higher than that of the control group (*p* = 0.038; Figure 3).

### Subgroup analyses for efficacy value

In the subgroup analyses, the viral clearance time of the following subgroups was significantly shorter in the molnupiravir group than in the control group: symptomatic, treatment time from first positive test <3 days, body mass index ≥24, and base- or booster-vaccinated subgroups (Table 3).

**TABLE 3:** Subgroup analysis of the differences in SARS-CoV-2 RNA shedding time using the non-parametric test.

Subgroup median (IQR)	Molnupiravir (n = 53)	Control (n = 58)	*p*-value
**Administration time since the first positive test**			
≥3ds	10 (6.0; 11.0) N = 21	10 (8.0; 10.0) N = 11	0.904
<3ds	9.50 (6.0; 10.5) N = 32	12 (8.5; 14.5) N = 47	0.000
**Gender**			
Female	10 (6.5; 11.5) N = 31	10 (8.0; 15.0) N = 26	0.087
Male	9.5 (5.0; 11.0) N = 22	11 (8.5; 12.5) N = 27	0.034
**BMI (Kg/m** ^**2**^ **)**			
≥24	8.0 (5.0; 10.0) N = 16	12 (9.0; 15.0) N = 17	0.005
<24	10 (7.0; 12.0) N = 37	10 (8.0; 12.0) N = 41	0.150
**Symptoms**			
Symptomatic	9 (6.0; 11.0) N = 46	11 (8.0; 14.0) N = 45	0.005
Asymptomatic	10 (9.0; 11.0) N = 7	10 (9.0; 12.0) N = 13	0.441
**Vaccination status**			
Unvaccinated	10.5 (8.0; 14.0) N = 6	12 (10.0; 13.0) N = 3	0.795
Base or booster vaccinated	9 (6.0; 11.0) N = 47	10 (8.0; 14.0) N = 55	0.003

SARS-CoV-2, severe acute respiratory syndrome coronavirus 2; IQR, interquartile range; BMI, body mass index;

^a^ Shedding time refers to the time since the first positive testing to the first day of nucleic acid ct value > 35 for both ORF1ab and N genes. Continuous variables were compared using the non-parametric test.

### Safety

Three adverse events were reported in the molnupiravir group. Two patients (3.77%) had diarrhea, and one patient (1.89%) had elevated levels of alanine aminotransferase. No adverse events of grade 3 or higher were reported, and no adverse events led to the discontinuation of molnupiravir treatment (Table 4).

**TABLE 4:** Summary of adverse events in the two groups.

Adverse event, n (%)	Molnupiravir (N = 53)	Control (N = 58)	*p*-value
Diarrhea	2 (3.77)	0 (0%)	1.000
Elevated ALT	1 (1.89)	0 (0%)	1.000

ALT, alanine aminotransferase.

## Discussion

Since the World Health Organization declared the Omicron variant as a variant of concern in November 2021, it has quickly become the dominant variant of SARS-CoV-2 worldwide because of its high contagiousness and high immune-escape ability caused by the high mutation of the spike protein. As a result of high mutations in the spike protein and RdRp, the Omicron variant may lead to reduced potency of existing RdRp inhibitors, such as molnupiravir, and successfully evade neutralizing antibodies (Islam et al., 2022; Mohsin and Mahmud, 2022; Takashita et al., 2022). In this randomized, controlled clinical trial, we evaluated the antiviral efficacy and adverse events of molnupiravir on patients with mild-to-moderate Omicron variant infection and found a statistically significant difference in the primary endpoint. Compared with the control group, the molnupiravir group had a significantly shorter viral clearance time of SARS-CoV-2 Omicron BA.2. No serious adverse events were reported.

Previous data on the efficacy and safety of molnupiravir in patients with COVID-19 mainly came from the MOVe-OUT trial (NCT04575597, MK-4482-002), and the major SARS-CoV-2 variants of infection were B.1.617.2 (Delta; 58.1%), B.1.621 (Mu; 20.5%), and P.1 (Gamma; 10.7%). All participants in the aforementioned study were not vaccinated against COVID-19 (Singh et al., 2022). In contrast to the participants in the MOVe-OUT trial, the main SARS-CoV-2 variant of infection in this study was Omicron BA.2, and 91.89% of the participants had been vaccinated against COVID-19. Our results showed that molnupiravir significantly accelerated viral clearance, as manifested by the significantly shorter median virus clearance time and the higher virus negativity rate at different time points after treatment of the molnupiravir group compared with the control group. These changes were consistent in the vaccine breakthrough-infected population (*p* = 0.003). Zou et al. (2022) obtained similar results in another study during the COVID-19 epidemic in Hong Kong in March 2022; their study included cases with a full vaccine course or a booster vaccine against COVID-19, and the main virus strain was the Omicron lineage. All these results demonstrated that molnupiravir retained its efficacy toward the Omicron variant *in vivo*, including the base- or booster-vaccinated populations.

With the evolution of the pathogenicity and infectivity of SARS-CoV-2, the clearance of viral RNA from patients and the interruption of viral transmission are critical to end the COVID-19 epidemic. Thus, this clinical trial was designed to assess the efficacy of molnupiravir on viral clearance rather than clinical endpoints, such as symptom duration or length of hospitalization. The patients included in this study were not limited to those at high risk of disease progression. The subgroup analyses demonstrated that the benefit of receiving molnupiravir treatment was evident among overweight and symptomatic patients. Furthermore, this study included asymptomatic cases (n = 20), e.g., positive for viral RNA but with no characteristic symptoms. However, compared with the controls, those treated with molnupiravir did not show any benefits (*p* = 0.441).

This study provides evidence to support the use of molnupiravir to accelerate viral RNA clearance during a COVID-19 epidemic dominated by the Omicron variant, potentially reducing the early transmission of the virus. However, several limitations remain. First, the sample size was relatively small, especially the number of asymptomatic cases, which limited our ability to fully characterize the efficacy of molnupiravir on asymptomatic patients. Second, this study could not be randomized and double-blinded because of the lack of a placebo in the control group; research deviation may have also occurred. Third, in view of the ethical issues, all the cases we included were under 70 years old. Therefore, here, we could not evaluate the efficacy of molnupiravir in elderly patients, but we specifically summarized the use experience of the elderly in another retrospective study (Liu et al., 2022).

In conclusion, a single-center, randomized, controlled trial was conducted in a population infected with the Omicron variant during the COVID-19 pandemic wave in Shanghai. The results of this study suggested that the administration of molnupiravir could safely shorten the viral clearance time, contribute to the implementation of the “dynamic zero-COVID-19” strategy, and accelerate the end of the epidemic.

## Data Availability

The original contributions presented in the study are included in the article/Supplementary Material; further inquiries can be directed to the corresponding authors.

## Abbreviations

BMI, body mass index; COVID-19, coronavirus disease 2019; RdRp, RNA-dependent RNA polymerase; SARS-CoV-2, severe acute respiratory syndrome coronavirus 2; SD, standard deviation; VL, viral load.
