# Supplementary material for: Efficacy and safety of molnupiravir in patients with Omicron variant vaccine breakthrough COVID-19 infection: a randomized, controlled trial
Source: Front Pharmacol. 2023 Nov 16;14:1197671. doi: 10.3389/fphar.2023.1197671 (PMC10687146; doi:10.3389/fphar.2023.1197671)
Supplement: Supplementary file 1 [file DataSheet1.docx]

Table S1. Summary of characteristics of real-world study of Molnupiravir

| No | **Participants** | Type of study | | | Country | Main clinical outcome | | Time | Reference |
| --- | --- | --- | --- | --- | --- | --- | --- | --- | --- |
| 1 | ●46 patients received molnupiravir  ●out-patients, mild and moderate forms of SARS-CoV-2 infection | Retrospective analysis | | | Romania | None of patients progressed to respiratory failure or required hospitalization | | Between February and March 2022 | [13] |
| 2 | ●5383 patients received molnupiravir and 49234 matched controls  ●non- hospitalized, aged 18 years or older, confirmed SARS-CoV-2 infection | Observational study | | | Hong Kong | Molnupiravir use was associated with lower risks of death and in-hospital disease progression than non-use, whereas risk of hospitalization was similar in both groups | | Between Feb 26 and June 26, 2022 | [23] |
| 3 | ●1856 patients received molnupiravir and 1856 matched controls  ●hospitalized, confirmed SARS-CoV-2 infection | Retrospective cohort study | | | Hong Kong | A lower risk of all-cause mortality was observed in Molnupiravir recipients versus matched controls | | Between Feb 26 and April 26, 2022 | [14] |
| 4 | ●180 patients were randomly assigned to receive either molnupiravir (n=90) or placebo (n=90)  ●adult (aged ≥18 years),outpatients, PCR-confirmed, mild-to-moderate SARS-CoV-2 infection | Randomized, placebo-controlled, double-blind, phase 2 trial (AGILECST-2) | | | UK | Participants in the molnupiravir group had a faster median time from randomization to negative PCR than participants in the placebo group. The probability of molnupiravir being superior to placebo (HR>1) was 75·4%, which was less than threshold of 80% | | Between Nov 18, 2020, and March 16, 2022, | [21] |
| 5 | ●molnupiravir users (n = 230) and non-users (n = 690)  ●hospitalized, mild-to-moderate COVID-19 | Retrospective study | | | Japan | The clinical deterioration rate was significantly lower in the molnupiravir users compared to the non-users | | Between January and April, 2022 | [15] |
| 6 | ●145 patients received molnupiravir  ●non-hospitalized, fully vaccinated, mild to moderate COVID-19 | Retrospective cohort study | | | Italy | 4 out of 145 patients (2.7%) required hospital admission, one patient (0.7%) required supplemental oxygen for medical conditions | | Between January 2022 and February 2022. | [24] |
| 7 | ●36 patients received molnupiravir  ●kidney transplant recipients and hemodialysis patients diagnosed with COVID-19 | Retrospective observational cohort study | | | Poland | Molnupiravir therapy can be safe and effective in the cohort of ESKD patients treated with hemodialysis or kidney transplantation | | Between February 2022 and April 2022 | [25] |
| 8 | ●168 patients received molnupiravir  ●aged ≥80 years, confirmed COVID-19 and mild-to-moderate illness | Retrospective single-center study | | | Italy | A total of 21 (12.5%) hospitalizations were reported at 28 days | | Between 11th January and 31st May 2022 | [26] |
| 9 | ●107 patients received molnupiravir  ●non-hospitalized, confirned SARS-CoV-2 infection | Prospective, observational single center study | | | Poland | Molnupiravir may constitute an alternative for certain subpopulations not eligible for the recommended first-line therapies, including immunocompromised patients and those with advanced-stage chronic kidney disease or hepatic injury | | Between  18 January 2022 and 31 March 2022 | [16] |
| 10 | ●45 patients received molnupiravir  ●hospitalized patients who developed SARS-CoV2-Infection | Retrospective study | | | Italy | 8 (18.1%) patients developed acute respiratory failure, and five (11.4%) patients died during hospitalisation | | 5 January to 5 March 2022 | [17] |
| 11 | ●100 patients received molnupiravir  ●mild or moderate, laboratory-confirmed COVID-19 | Retrospective, single-center cohort study | | | Italy | Participants treated with molnupiravir demonstrated early clinical improvement, no need for hospitalization. | | 8 January to 24 April 2022 | [18] |
| 12 | ● 12529 participants from the molnupiravir plus usual care group, and 12525 from the usual care group  ●out-patient with SARS-CoV-2 infection | Open-label, multigroup, prospective, platform adaptive trial (PANORAMIC) | | | UK | Molnupiravir did not reduce the frequency of COVID-19-associated hospitalisations or death among high-risk vaccinated adults in the community. | | Between Dec 8, 2021, and April 27, 2022 | [22] |
| 13 | ●203 patients received molnupiravir, 387 did not receive any antiviral regimen  ●hospitalized patient | Retrospective study | | | Poland | Treatment with molnupiravir resulted in a statistically significant reduction in mortality during the 28-day follow-up (9.9 vs. 16.3%). | | Between 1 January and 30 April 2022 | [19] |
| 14 | - 26 patients received molnupiravir and 16 from the standard care group - Age≥60 years old, hospitalized, PCR confirmed SARS-CoV-2 infection | | Retrospective study | China | | Early initiation of molnupiravir in elderly COVID-19 patients with omicron B A.2, but was not associated with low risk of disease progression. | From 26 March to 31 May 2022 | | [20] |
